# Additive risk survival model with microarray data

**DOI:** 10.1186/1471-2105-8-192

**Published:** 2007-06-08

**Authors:** Shuangge Ma, Jian Huang

**Affiliations:** 1 Department of Epidemiology and Public Health, Yale University, New Haven, CT 06520, USA; 2 Department of Statistics and Actuarial Science, University of Iowa, Iowa City, IA 52242, USA

## Abstract

**Background:**

Microarray techniques survey gene expressions on a global scale. Extensive biomedical studies have been designed to discover subsets of genes that are associated with survival risks for diseases such as lymphoma and construct predictive models using those selected genes. In this article, we investigate simultaneous estimation and gene selection with right censored survival data and high dimensional gene expression measurements.

**Results:**

We model the survival time using the additive risk model, which provides a useful alternative to the proportional hazards model and is adopted when the absolute effects, instead of the relative effects, of multiple predictors on the hazard function are of interest. A Lasso (least absolute shrinkage and selection operator) type estimate is proposed for simultaneous estimation and gene selection. Tuning parameter is selected using the V-fold cross validation. We propose Leave-One-Out cross validation based methods for evaluating the relative stability of individual genes and overall prediction significance.

**Conclusion:**

We analyze the MCL and DLBCL data using the proposed approach. A small number of probes represented on the microarrays are identified, most of which have sound biological implications in lymphoma development. The selected probes are relatively stable and the proposed approach has overall satisfactory prediction power.

## Background

Microarray techniques provide a way of monitoring gene expressions on a global scale. An important application of microarray is to discover subsets of genes that are associated with occurrence of certain diseases, for example breast cancer, leukemia or lymphoma. Biomedical studies have been conducted to measure gene expression levels and patients' survival information simultaneously. This article is partly motivated by studies like the Mantle Cell Lymphoma (MCL) study reported in [1], where expression levels of 8810 genes and survival information are measured for 92 subjects. A main goal of the MCL study is to discover subsets of genes that are linked with patients' survival risk. Statistically, standard estimation approaches cannot yield a unique estimator, when the dimension of gene expressions is greater than the sample size. Biologically, it is reasonable to assume that only a small number of genes are relevant to predicting the lymphoma occurrence. It is thus of great interest to develop statistical methodologies that can carry out simultaneous estimation and dimension reduction or variable selection.

Dimension reduction has been extensively investigated for linear regression models [2,3]. One widely used approach is to use low dimensional projections of the covariates (genes) as surrogates for the true covariates. Examples include the ridge regression, partial least squares (PLS), and principal component regression (PCR). For a detailed discussion, see [2]. Including all the covariates in the predictive models through projections may introduce noises and lead to poor predictive performance. In addition, the underlying assumption that all covariates are associated with the disease progression is not necessarily true.

An alternative approach is to use variable selection techniques and identify the important covariates. This can usually be accomplished by using penalization methods, where penalties with data dependent tuning parameters are used to control the sparsity of the models. A general scheme is outlined in [4]. Penalization methods are especially effective when there exist a small number of large covariate effects. Compared with projection approaches, penalized variable selection may be preferred in microarray studies since it is capable of selecting a small number of individual covariates and only those covariates are used in the predictive models. Further biological investigation can focus on those identified influential genes only.

Modeling survival outcome with high dimensional gene expressions is more challenging due to the presence of censoring and the use of complicated semiparametric models. One approach used by [5] is to cluster genes first, and then use the sample averages of the gene expression levels in a Cox model for right censored survival outcome. Another well developed clustering based algorithm is the gene harvesting procedure of [6]. Standard PLS method has been employed in [7], where the resulted PLS components are used in the Cox model. A penalized estimation procedure has been considered for the Cox model using kernels [8], under the assumption that the covariate effects are smooth functions of gene expression levels. The Lasso (least absolute shrinkage and selection) type estimator in the Cox model with right censored data has been proposed [9], and [10] applies the LARS algorithm in computing the Lasso estimator in the Cox model. In a recent study, [11] applies the principal component regression to additive risk models with right censored data. Aforementioned studies, except [11], assume the proportional hazards models for survival time. Although this is the most straightforward assumption, it is not necessarily satisfied.

Survival analysis assuming the additive risk model may provide more insights beyond the proportional hazards model analysis. An additive model is generally adopted when it is reasonable to assume that the covariate effects under consideration contribute additively to the conditional hazard. Consider one special form of the additive risk models investigated in [12], where we model the conditional hazard at time *t *by

λ(t|Z)=λ0(t)+β′0Z,
 MathType@MTEF@5@5@+=feaafiart1ev1aaatCvAUfKttLearuWrP9MDH5MBPbIqV92AaeXatLxBI9gBaebbnrfifHhDYfgasaacH8akY=wiFfYdH8Gipec8Eeeu0xXdbba9frFj0=OqFfea0dXdd9vqai=hGuQ8kuc9pgc9s8qqaq=dirpe0xb9q8qiLsFr0=vr0=vr0dc8meaabaqaciaacaGaaeqabaqabeGadaaakeaaiiGacqWF7oaBcqGGOaakcqWG0baDcqGG8baFcqWGAbGwcqGGPaqkcqGH9aqpcqWF7oaBdaWgaaWcbaGaeGimaadabeaakiabcIcaOiabdsha0jabcMcaPiabgUcaRiqb=j7aIzaafaWaaSbaaSqaaiabicdaWaqabaGccqWGAbGwcqGGSaalaaa@410E@

given a *d*-dimensional vector of time-independent covariates *Z*. Here *β*_0 _and *λ*_0_(·) denote the unknown regression parameter and the unknown baseline hazard function, respectively. Previous studies have concluded its sound biological and empirical basis [13] and satisfactory statistical properties [12,14].

The objective function under the additive risk model proposed in [12] takes a least-squares form, which makes estimation computationally easier than under the proportional hazards model. This is especially important for high dimensional microarray studies where computation cost is a serious concern. Inspired by the special form of the objective function, we propose a Lasso approach for estimation and gene selection in the additive risk model (1), which minimizes a least-squares objective function subject to a *L*_1 _constraint, when high dimensional microarray measurements are present. Because of the nature of the *L*_1 _constraint, the Lasso method shrinks coefficients and sets some estimated coefficients exactly zero. Thus it can yield a sparse model.

In this article, we investigate survival analysis with high dimensional gene expressions under the additive risk model. The goal is to provide an alternative estimation and gene selection method, when the proportional hazard assumption cannot be satisfied. Beyond modeling the survival risk in an additive manner, the proposed approach is computationally affordable and well behaved. Evaluation of stability of individual genes and overall predictive power, which has been ignored in some of previous studies, are also investigated.

## Results

### MCL data

A study using microarray expression analysis of mantle cell lymphoma (MCL) is reported in [1]. The primary goal of this study is to discover genes that have good predictive power of patients' survival risk. Among 101 untreated patients with no history of previous lymphoma included in this study, 92 were classified as having MCL, based on established morphologic and immunophenotypic criteria. Survival times of 64 patients were available and other 28 patients were censored. The median survival time was 2.8 years (range 0.02 to 14.05 years). Lymphochip DNA microarrays [5] were used to quantify mRNA expressions in the lymphoma samples from the 92 patients. The gene expression data that contains expression values of 8810 cDNA elements (probes) is available at [15].

We use the additive risk model (1) for the conditional hazards given the gene expression values and apply the proposed Lasso method. Although there is no theoretical or numerical limitation on the number of probes that can be used in the proposed approach, we pre-process the data as follows to exclude noises and gain further stability: (1) Un-supervised screening: compute the interquartile ranges of all gene expressions. Remove the probes with interquartile ranges smaller than their first quartile. 6608 probes pass this screening; (2) Supervised screening: compute correlation coefficients of the uncensored survival times with gene expressions. Select the 500 probes with the largest absolute values of the correlation coefficients. We refer to [16] for more discussions of gene screening. We then standardize these 500 probes to have mean 0 and variance 1.

**Figure 1:**
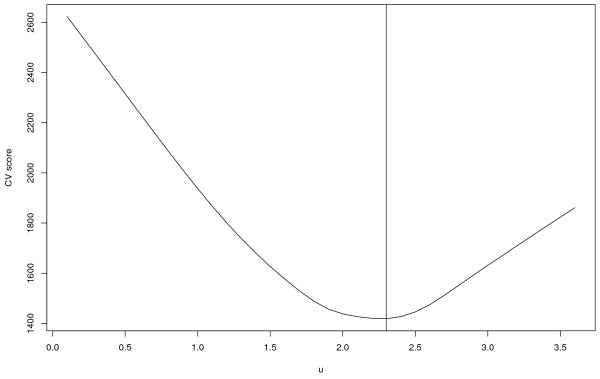
MCL data: CV score as a function of tuning parameter *u*.

In applying the proposed Lasso approach, we select the tuning parameter *u *via the five-fold cross validation. We show in Figure 1 the CV score as a function of the tuning parameter *u*. There is a well-defined minimum at *u *= 2.3. Using this cross validated tuning parameter, only 6 out of 500 probes have nonzero estimated coefficients in the predictive model. We show in Table 1 the list of those six probes, their official symbols, estimates and corresponding OI (occurrence index). Two of these six probes, UNIQID 23826 and 34790, are from the same gene TK1.

**Table 1:** MCL data: genes with nonzero estimates.

UNIQID	Official Symbol	Gene Name	Estimate	OI
17149	RAD54L	Hs.523220, RAD54-like (S. cerevisiae)	0.287	0.967
17691	SOCS1	Hs.50640, Suppressor of cytokine signaling 1	0.104	0.228
17821	CDKN1C	Hs.106070, Cyclin-dependent kinase inhibitor 1C	0.286	0.978
23826	TK1	Hs.515122, Thymidine kinase 1, soluble	0.678	0.967
32699	UHRF1	Hs.108106, Ubiquitin-like, containing PHD	0.229	0.902
		and RING finger domains, 1		
34790	TK1	Hs.515122, Thymidine kinase 1, soluble	0.709	0.967

The descriptions of the six probes can be found at the NCBI website [17]. RAD54 (gene Hs.523220) is highly expressed in organs of germ cells and lymphoid development. The protein encoded by this gene has been shown to play a role in homologous recombination related repair of DNA double-strand breaks. Defects in RAD54L may be a cause of tumor formation. Mutations of the tumor suppressor gene SOCS-1 (Hs.50640) in classical Hodgkin lymphoma are frequent and associated with nuclear phospho-STAT5 accumulation. Methylation of the SOCS-1 gene is associated with lymph node metastasis, advanced tumor stage and reduced expression of SOCS-1 in GC tissues. It has also been shown that SOCS-1 stringently regulates development and homeostasis of interleukin-7 and interleukin-15 in T lymphocyte. In lymphoma, decreased expression of p57 (Hs.106070) has been observed in about 50% of cases. p57 plays a role in negatively regulating the cell proliferation of thyroid lymphoma cells and decreased expression of it contributes to the progression of the disease. Aberrant DNA methylation of this gene occurs in the promoter region in lymphoid malignancies of B-cell phenotype Peripheral blood TK1 (Hs.515122, UNIQID 23826 and 34790) isozyme is a useful independent biochemical marker for the subgroup of adult non-Hodgkin's lymphoma who respond poorly to current therapy [18]. In a clinical study, it is shown that long-term treatment of H9 human lymphoid cells in the presence of dideoxycytidine may down-regulate TK1 gene expression. The RING domain of UHRF1 (Hs.108106) is a functional determinant of growth regulation and UHRF1 is generally required in tumor cell proliferation.

All six probes are either directly associated with lymphoma or important in tumor proliferation. The encouraging discovery results partly support the effectiveness of the proposed additive model and Lasso approach. Out of the six probes in Table 1, two (UNIQID: 32699, 34790) are also identified in [1]. We note that the difference between the sets of probes identified is at least partly caused by the difference in model assumptions. In our study, an additive risk model is assumed, whereas in [1] a Cox model assumption is made.

Evaluation is carried out using the Leave-One-Out (LOO) approach proposed in the Methods section. Note that for each reduced dataset (with one subject removed), we carry out the supervised screening and select possibly different set of 500 probes out of the probes that pass the unsupervised screening. Since the unsupervised screening is mainly due to technical considerations and the survival outcome is not used in the selection, the evaluation results are expected to accurately reflect the stability and prediction power.

**Figure 2:**
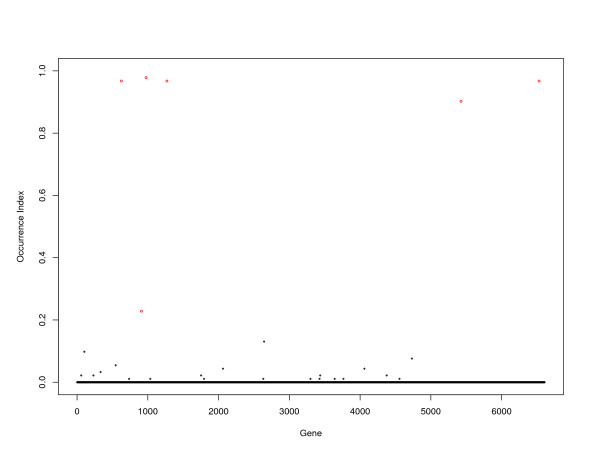
MCL data: occurrence index of individual genes.

The OIs of individual probes are computed and shown in Figure 2. In Figure 2, red dots represent the six probes identified using the proposed approach; while black dots represent the rest 6603 genes. We can see that genes identified via Lasso have larger OIs than the rest probes, indicating greater importance. This also shows that the proposed approach is relatively stable. We see from Table 1 that five out of those six probes have OIs close to 1. Gene SOCS-1 has smaller OI (0.228), but it is still larger than OIs for the unselected probes.

**Figure 3:**
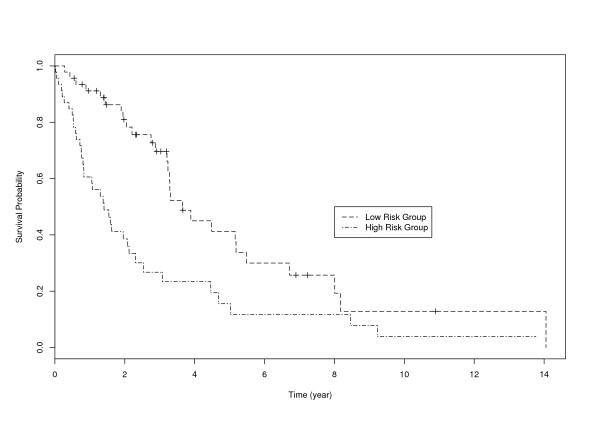
MCL data: survival functions of two hypothetical risk groups.

The overall predictive performance is also evaluated using the LOO approach. We generate two hypothetical risk groups of equal sizes based on the predictive risk scores. We show in Figure 3 survival functions of the two risk groups. We can clearly see that the two survival functions differ significantly. Chi-square test of the difference yields test statistic 13.0 and p-value 0.0003. So we can conclude that the proposed approach can satisfactorily predict survival risk based on selected gene expression measurements.

To our best knowledge, the only available published article dealing with additive risk model and high dimensional gene measurements is [11], where the PCR is used for model reduction. Since the PCR is not a variable selection method, it is not directly comparable to the proposed Lasso approach. Hence we only consider the following simple alternative approach for comparison. We identify ten probes with marginally largest (absolute values of) correlation coefficients with the event time for uncensored subjects. Ten probes are selected so that the degree of freedom is slightly larger than but roughly matches the Lasso approach. Predictive models are constructed using those ten probes only. We compute the predictive power using the proposed LOO based approach and obtain a Chi-square test statistic 10.9, which is also significant with p-value 0.001. The difference between the two risk groups is smaller than that from the proposed approach, which suggests less predictive power.

We note that in Table 1 two probes (UNIQID 23826 and 34790) correspond to the same gene TK1. It has been suggested in literature that analysis can be carried out at the gene level by combining measurements from two or multiple probes. However, we find that the correlations of two (or multiple) probes from the same genes in the MCL data can be low. So it is not clear whether it is proper to simply take a summary measure such as the average of the two probes or arbitrarily discard one of them in the analysis. In our study, we follow [1] and carry out the analysis at the probe level.

### DLBCL data

The DLBCL (diffuse large B-cell lymphoma) data was first analyzed in [19]. This dataset consists of 240 patients with DLBCL, including 138 patient deaths during the followup. Expression profiles of 7399 probes are obtained. Missing values are imputed using a nearest neighbor approach as in [8]. We carry out supervised selection and select 500 probes with the largest absolute values of marginal correlation coefficients with the uncensored event times to gain further stability. Probes are then normalized to have zero mean and unit variance.

Five-fold cross validation is used to determine the optimal tuning parameter. A plot (omitted here) similar to Figure 1 yields optimal *u *= 0.7. With the optimal *u*, 17 probes have nonzero estimated coeficients in the predictive model. For the genes corresponding to these probes, their official symbols, descriptions, estimated coefficients and corresponding OIs are shown in Table 2.

**Table 2:** DLBCL data: genes with nonzero estimates.

UNIQID	Official Symbol	Gene Name	Estimate	OI
27774	CDK7	Hs.184298, cyclin-dependent kinase 7	-0.045	0.333
26627	SMYD5	Hs.54413, retinoic acid induced 15	0.023	0.604
27556	CPR2	Hs.347349, cell cycle progression 2 protein	0.029	0.017
16804			0.015	0.004
31561	LDHA	Hs.2795, lactate dehydrogenase A	-0.009	0.346
16480	NME1	Hs.118638, non-metastatic cells 1,	0.036	0.938
		protein (NM23A) expressed i		
29610	IGFALS	Hs.839, insulin-like growth factor binding protein,	0.006	0.013
		acid labile subunit		
28383	CR2	Hs.73792, complement component receptor 2	-0.013	0.054
17804	HLA-DMB	Hs.77522, major histocompatibility complex,	-0.032	0.021
		class II, DM alpha		
17280	HLA-DRB1	Hs.73931, major histocompatibility complex,	-0.013	0.004
		class II, DQ beta 1		
16359	PTK2	Hs.740, PTK2 protein tyrosine kinase 2	-0.041	0.225
29650	MFHAS1	Hs.24724, malignant fibrous histiocytoma	-0.040	0.379
		amplified sequence 1		
27593	MMP2	Hs.111301, matrix metalloproteinase 2	-0.021	0.000
16754	MMP2	Hs.111301, matrix metalloproteinase 2	-0.012	0.358
15937	ST14	Hs.119222, suppression of tumorigenicity 13	0.012	0.104
		(colon carcinoma)		
28840	EDN1	Hs.2271, endothelin 1	0.019	0.000
30130		Hs.96557, Homo sapiens cDNA FLJ12727 fis,	0.010	0.000
		clone NT2RP2000027		

Five probes in Table 2 (UNIQID: 27556, 16804, 31561, 16480 and 29610) are also identified in [19]. Quite a few probes we identify have been confirmed to be associated with lymphoma by other independent studies. The level of Lactose Dehydrogenase (LDH, Hs.2795) can go high in reaction to many different kinds of stress or damage to body tissues. As tissues are damaged they release more LDH. Although an LDH test isn't very useful for an initial diagnosis of lymphoma it is frequently used as monitoring test for those who already have lymphoma. Any elevation from the normal range may indicate a relapse or renewed growth. The probe from the gene Hs.118638 (official symbol NME1) has been shown to play an important role in development of DLBCL [20]. Abnormal expression of NM23 is associated with malignant potential, lymph node metastasis and clinical stage, and it may play a role in development of gastric cancer. Complement component receptor-2 (CR2, Hs.73792) is the membrane protein on B lymphocytes to which the Epstein-Barr virus (EBV) binds during infection of these cells. EBV/C3d receptor interacts with the p53 anti-oncoprotein expressed in the human B lymphoma cells. HLA-DMA (Hs.77522) and HLA-DQB1 (Hs.73931) belong to the HLA class II alpha chain paralogues. This class II molecule is a heterodimer consisting of an alpha (DMA) and a beta chain (DMB), both anchored in the membrane. It plays a central role in the immune system by presenting peptides derived from extracellular proteins. Class II molecules are expressed in antigen presenting cells (APC: B lymphocytes, dendritic cells, macrophages). The probe corresponding to the Gene Hs.24724 (official symbol MFHAS1, also known as MASL1) is a candidate oncogene found in amplification of 8p23.1 and translocated in an immunoblastic B-cell lymphoma cell line. MMP-2 (Hs.111301) expression has significant correlation with tumor invasion, tumor differentiation and lymph node metastases; MMP-2 may participate in the development of lymph node micrometastasis of gastric carcinoma. Strong MMP-2 expression is correlated with a favorable prognosis of Hodgkin's lymphoma.

**Figure 4:**
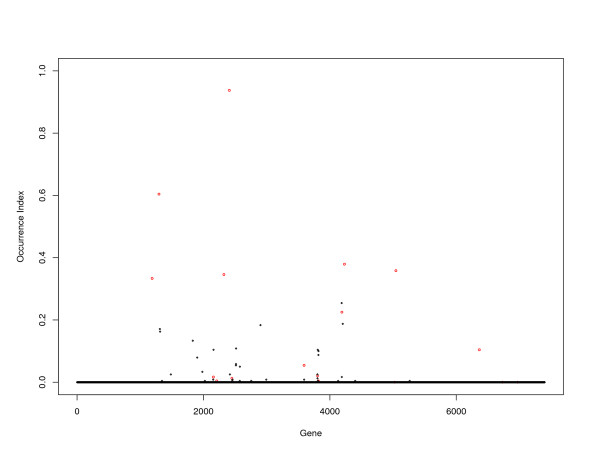
DLBCL data: occurrence index of individual genes.

Stability of individual probes is evaluated using the proposed OI. Figure 4 shows the OI computed from the LOO approach. We can see that several genes we identify have larger OIs than the rest. However, some identified genes have OIs close to zero. On the other hand, there are a few probes with large OIs but are not included in the predictive model. Figure 4 suggests that the "signals" in the DLBCL data are not as strong as in the MCL data and the final predictive model is less reliable. We note that with a relatively small sample size and large number of probes, the randomness caused by the finite fold cross validation is not ignorable. This partly accounts for the small OIs for some identified genes.

For probes with small OIs: it is still not clear what the biological functions are for the gene with UNIQID 16804 (OI = 0.000); Gene Hs.73931 has similar functions as Hs.77522. With the Lasso approach, only one gene (from a highly co-regulated gene group) tends to be selected. This partly explains why OI for Hs.73791 is much smaller than that of Hs.77522. Similar explanation holds for the OI of gene Hs.111301 (UNIQID 27593); For genes Hs.2271 and Hs.96557, there are documented studies showing they are associated with lymphoma or in general tumor progressions.

The overall prediction power of the proposed approach is assessed with the approach described in the Methods section. The Chi-square statistic (for testing the difference between the two hypothetical risk groups) is 11.2, with p-value 0.0008. We thus conclude that the proposed approach can effectively predict survival risks based on a small subset of selected genes. As an alternative, the simple approach with 20 marginally most important probes yields Chi-square statistic 1.8 (with p-value 0.176). So unlike the MCL data, the simple approach cannot predict survival risk based on marginally selected genes.

## Conclusion

It is of great practical interest to develop sound statistical techniques that are capable of identifying a small subset of influential genes and predicting survival risks based on selected genes. In this article, we assume the additive risk model for survival times and propose using Lasso for simultaneous estimation and gene selection. This is the first attempt to use the additive risk model and penalized variable selection in microarray survival study. Evaluation of individual genes and overall prediction performance are based on the LOO techniques. We analyze the MCL and DLBCL data using the proposed approach. Empirical studies show that the proposed approach can identify a small subset of genes with satisfactory prediction performance. Most of the genes we identify have been shown to be associated with lymphoma in other studies. The effectiveness of the additive model and the Lasso method is supported by the satisfactory prediction performance, reasonable gene discovery results and the correspondence between the OI and published gene discovery results.

In this article, additive risk model is assumed, whereas in most previous studies, Cox model assumption is made. In the additive model, we assume the genes contribute to the survival hazard in an additive manner. So the estimation results may be easier to explain than those from the Cox model. Possible alternative survival models include the accelerated failure time and accelerated hazard models. Each model has its own advantages and is preferred under different circumstances. With high dimensional microarray measurements, it is still an open question how to compare different survival model fitting results. Such a comparison study is beyond the scope of this article.

With the proposed Lasso approach, we are able to identify individual gene effects. This advantage is not shared by approaches like the principal component analysis or the partial least squares. However, the tradeoff is that the number of gene effects can be evaluated is limited by the sample size. If it is biologically reasonable to believe that the number of genes significantly associated with survival is larger than the sample size, then transformation of the gene expressions will be firstly needed. One possibility is to use linear combinations of individual gene expressions as covariates. We postpone pursuing this to a separate study.

## Methods

### Additive risk model

Consider a set of *n *independent observations (*T*_*i*_, *C*_*i*_, *Z*_*i*_), *i *= 1, ..., *n*. Suppose that the *i*^*th *^subject's event time *T*_*i *_is conditionally independent of the censoring time *C*_*i*_, given the *d*-dimensional covariate vector *Z*_*i*_. For simplicity of notations, we consider time-independent *Z *only throughout this paper unless otherwise specified. Let *X*_*i *_= *min*(*T*_*i*_, *C*_*i*_) and *δ*_*i *_= *I*(*T*_*i *_≤ *C*_*i*_) for right censored data. We assume the additive risk model (1). Other format of additive risk models have been studied in [14]. For the *i*^*th *^subject, denote {*N*_*i*_(*t*) = *I*(*X*_*i *_≤ *t*, *δ*_*i *_= 1); *t *≥ 0} and {*Y*_*i*_(*t*) = *I*(*X*_*i *_≥ *t*); *t *≥ 0} as the observed event process and the at-risk process, respectively. The regression coefficient *β*_0 _can be estimated by solving the following estimating equation:

U(β)=∑i=1n∫0∞Zi{dNi(t)−Yi(t)dΛ^(β,t)−Yi(t)β′Zidt}=0,
 MathType@MTEF@5@5@+=feaafiart1ev1aaatCvAUfKttLearuWrP9MDH5MBPbIqV92AaeXatLxBI9gBaebbnrfifHhDYfgasaacH8akY=wiFfYdH8Gipec8Eeeu0xXdbba9frFj0=OqFfea0dXdd9vqai=hGuQ8kuc9pgc9s8qqaq=dirpe0xb9q8qiLsFr0=vr0=vr0dc8meaabaqaciaacaGaaeqabaqabeGadaaakeaacqWGvbqvcqGGOaakiiGacqWFYoGycqGGPaqkcqGH9aqpdaaeWbqaamaapedabaGaemOwaO1aaSbaaSqaaiabdMgaPbqabaGccqGG7bWEcqWGKbazcqWGobGtdaWgaaWcbaGaemyAaKgabeaakiabcIcaOiabdsha0jabcMcaPiabgkHiTiabdMfaznaaBaaaleaacqWGPbqAaeqaaOGaeiikaGIaemiDaqNaeiykaKIaemizaqMafu4MdWKbaKaacqGGOaakcqWFYoGycqGGSaalcqWG0baDcqGGPaqkcqGHsislcqWGzbqwdaWgaaWcbaGaemyAaKgabeaakiabcIcaOiabdsha0jabcMcaPiqb=j7aIzaafaGaemOwaO1aaSbaaSqaaiabdMgaPbqabaGccqWGKbazcqWG0baDcqGG9bqFcqGH9aqpcqaIWaamaSqaaiabicdaWaqaaiabg6HiLcqdcqGHRiI8aOGaeiilaWcaleaacqWGPbqAcqGH9aqpcqaIXaqmaeaacqWGUbGBa0GaeyyeIuoaaaa@6AFA@

where Λ^
 MathType@MTEF@5@5@+=feaafiart1ev1aaatCvAUfKttLearuWrP9MDH5MBPbIqV92AaeXatLxBI9gBaebbnrfifHhDYfgasaacH8akY=wiFfYdH8Gipec8Eeeu0xXdbba9frFj0=OqFfea0dXdd9vqai=hGuQ8kuc9pgc9s8qqaq=dirpe0xb9q8qiLsFr0=vr0=vr0dc8meaabaqaciaacaGaaeqabaqabeGadaaakeaacuqHBoatgaqcaaaa@2E31@(*β*, *t*) is the estimator of Λ_0 _satisfying

Λ^(β^,t)=∫0∞{dNi(u)−Yi(u)β^′Zidu}∑i=1nYi(u).
 MathType@MTEF@5@5@+=feaafiart1ev1aaatCvAUfKttLearuWrP9MDH5MBPbIqV92AaeXatLxBI9gBaebbnrfifHhDYfgasaacH8akY=wiFfYdH8Gipec8Eeeu0xXdbba9frFj0=OqFfea0dXdd9vqai=hGuQ8kuc9pgc9s8qqaq=dirpe0xb9q8qiLsFr0=vr0=vr0dc8meaabaqaciaacaGaaeqabaqabeGadaaakeaacuqHBoatgaqcaiabcIcaOGGaciqb=j7aIzaajaGaeiilaWIaemiDaqNaeiykaKIaeyypa0Zaa8qmaeaadaWcaaqaaiabcUha7jabdsgaKjabd6eaonaaBaaaleaacqWGPbqAaeqaaOGaeiikaGIaemyDauNaeiykaKIaeyOeI0IaemywaK1aaSbaaSqaaiabdMgaPbqabaGccqGGOaakcqWG1bqDcqGGPaqkcuWFYoGygaqcgaqbaiabdQfaAnaaBaaaleaacqWGPbqAaeqaaOGaemizaqMaemyDauNaeiyFa0habaWaaabmaeaacqWGzbqwdaWgaaWcbaGaemyAaKgabeaakiabcIcaOiabdwha1jabcMcaPaWcbaGaemyAaKMaeyypa0JaeGymaedabaGaemOBa4ganiabggHiLdaaaaWcbaGaeGimaadabaGaeyOhIukaniabgUIiYdGccqGGUaGlaaa@5F74@

The resulting estimator of *β*_0 _satisfies the simple estimating equation

[∑i=1n∫0∞Yi(t){Zi−Z¯(t)}⊗2dt]β^=[∑i=1n∫0∞{Zi−Z¯(t)}dNi(t)],
 MathType@MTEF@5@5@+=feaafiart1ev1aaatCvAUfKttLearuWrP9MDH5MBPbIqV92AaeXatLxBI9gBaebbnrfifHhDYfgasaacH8akY=wiFfYdH8Gipec8Eeeu0xXdbba9frFj0=OqFfea0dXdd9vqai=hGuQ8kuc9pgc9s8qqaq=dirpe0xb9q8qiLsFr0=vr0=vr0dc8meaabaqaciaacaGaaeqabaqabeGadaaakeaadaWadaqaamaaqahabaWaa8qmaeaacqWGzbqwdaWgaaWcbaGaemyAaKgabeaakiabcIcaOiabdsha0jabcMcaPiabcUha7jabdQfaAnaaBaaaleaacqWGPbqAaeqaaOGaeyOeI0IafmOwaOLbaebacqGGOaakcqWG0baDcqGGPaqkcqGG9bqFdaahaaWcbeqaaiabgEPielabikdaYaaakiabdsgaKjabdsha0bWcbaGaeGimaadabaGaeyOhIukaniabgUIiYdaaleaacqWGPbqAcqGH9aqpcqaIXaqmaeaacqWGUbGBa0GaeyyeIuoaaOGaay5waiaaw2faaGGaciqb=j7aIzaajaGaeyypa0ZaamWaaeaadaaeWbqaamaapedabaGaei4EaSNaemOwaO1aaSbaaSqaaiabdMgaPbqabaGccqGHsislcuWGAbGwgaqeaiabcIcaOiabdsha0jabcMcaPiabc2ha9jabdsgaKjabd6eaonaaBaaaleaacqWGPbqAaeqaaOGaeiikaGIaemiDaqNaeiykaKcaleaacqaIWaamaeaacqGHEisPa0Gaey4kIipaaSqaaiabdMgaPjabg2da9iabigdaXaqaaiabd6gaUbqdcqGHris5aaGccaGLBbGaayzxaaGaeiilaWcaaa@74B5@

where Z¯(t)=∑i=1nYi(t)Zi/∑i=1nYi(t)
 MathType@MTEF@5@5@+=feaafiart1ev1aaatCvAUfKttLearuWrP9MDH5MBPbIqV92AaeXatLxBI9gBaebbnrfifHhDYfgasaacH8akY=wiFfYdH8Gipec8Eeeu0xXdbba9frFj0=OqFfea0dXdd9vqai=hGuQ8kuc9pgc9s8qqaq=dirpe0xb9q8qiLsFr0=vr0=vr0dc8meaabaqaciaacaGaaeqabaqabeGadaaakeaacuWGAbGwgaqeaiabcIcaOiabdsha0jabcMcaPiabg2da9maaqadabaGaemywaK1aaSbaaSqaaiabdMgaPbqabaGccqGGOaakcqWG0baDcqGGPaqkcqWGAbGwdaWgaaWcbaGaemyAaKgabeaaaeaacqWGPbqAcqGH9aqpcqaIXaqmaeaacqWGUbGBa0GaeyyeIuoakiabc+caVmaaqadabaGaemywaK1aaSbaaSqaaiabdMgaPbqabaGccqGGOaakcqWG0baDcqGGPaqkaSqaaiabdMgaPjabg2da9iabigdaXaqaaiabd6gaUbqdcqGHris5aaaa@4F21@. Denote

Li=∫0∞Yi(t){Zi−Z¯(t)}⊗2dt,Ri=∫0∞{Zi−Z¯(t)}dNi(t).
 MathType@MTEF@5@5@+=feaafiart1ev1aaatCvAUfKttLearuWrP9MDH5MBPbIqV92AaeXatLxBI9gBaebbnrfifHhDYfgasaacH8akY=wiFfYdH8Gipec8Eeeu0xXdbba9frFj0=OqFfea0dXdd9vqai=hGuQ8kuc9pgc9s8qqaq=dirpe0xb9q8qiLsFr0=vr0=vr0dc8meaabaqaciaacaGaaeqabaqabeGadaaakeaafaqabeqacaaabaGaemitaW0aaWbaaSqabeaacqWGPbqAaaGccqGH9aqpdaWdXaqaaiabdMfaznaaBaaaleaacqWGPbqAaeqaaOGaeiikaGIaemiDaqNaeiykaKcaleaacqaIWaamaeaacqGHEisPa0Gaey4kIipakiabcUha7jabdQfaAnaaBaaaleaacqWGPbqAaeqaaOGaeyOeI0IafmOwaOLbaebacqGGOaakcqWG0baDcqGGPaqkcqGG9bqFdaahaaWcbeqaaiabgEPielabikdaYaaakiabdsgaKjabdsha0jabcYcaSaqaaiabdkfasnaaCaaaleqabaGaemyAaKgaaOGaeyypa0Zaa8qmaeaacqGG7bWEcqWGAbGwdaWgaaWcbaGaemyAaKgabeaakiabgkHiTiqbdQfaAzaaraGaeiikaGIaemiDaqNaeiykaKIaeiyFa0NaemizaqMaemOta40aaSbaaSqaaiabdMgaPbqabaGccqGGOaakcqWG0baDcqGGPaqkaSqaaiabicdaWaqaaiabg6HiLcqdcqGHRiI8aaaakiabc6caUaaa@6892@

*L*^*i*^s are symmetric semi-positive-definite matrices with rank equal to 1.

In a typical microarray study such as the MCL, we usually have *d *~ 10^3 ^- 10^4^, while the number of subjects *n *is at most ~10^2^. In this case, the left hand side of (4) does not have full rank, so a unique solution to equation (4) does not exist. Certain regularization or model reduction will be needed along with estimation. Especially, we propose using the Lasso regularization.

### The Lasso method

Denote the (*s, l*) element of *L*^*i *^as Ls,li
 MathType@MTEF@5@5@+=feaafiart1ev1aaatCvAUfKttLearuWrP9MDH5MBPbIqV92AaeXatLxBI9gBaebbnrfifHhDYfgasaacH8akY=wiFfYdH8Gipec8Eeeu0xXdbba9frFj0=OqFfea0dXdd9vqai=hGuQ8kuc9pgc9s8qqaq=dirpe0xb9q8qiLsFr0=vr0=vr0dc8meaabaqaciaacaGaaeqabaqabeGadaaakeaacqWGmbatdaqhaaWcbaGaem4CamNaeiilaWIaemiBaWgabaGaemyAaKgaaaaa@3305@ and the *s*^*th *^component of *R*^*i *^and *β *as Rsi
 MathType@MTEF@5@5@+=feaafiart1ev1aaatCvAUfKttLearuWrP9MDH5MBPbIqV92AaeXatLxBI9gBaebbnrfifHhDYfgasaacH8akY=wiFfYdH8Gipec8Eeeu0xXdbba9frFj0=OqFfea0dXdd9vqai=hGuQ8kuc9pgc9s8qqaq=dirpe0xb9q8qiLsFr0=vr0=vr0dc8meaabaqaciaacaGaaeqabaqabeGadaaakeaacqWGsbGudaqhaaWcbaGaem4CamhabaGaemyAaKgaaaaa@30D0@ and *β*_*s*_, respectively. We can see that equation (4) is equivalent to the following *d *equations:

(∑i=1nLs,1i)β1+...+(∑i=1nLs,di)βd=∑i=1nRsi,
 MathType@MTEF@5@5@+=feaafiart1ev1aaatCvAUfKttLearuWrP9MDH5MBPbIqV92AaeXatLxBI9gBaebbnrfifHhDYfgasaacH8akY=wiFfYdH8Gipec8Eeeu0xXdbba9frFj0=OqFfea0dXdd9vqai=hGuQ8kuc9pgc9s8qqaq=dirpe0xb9q8qiLsFr0=vr0=vr0dc8meaabaqaciaacaGaaeqabaqabeGadaaakeaadaqadaqaamaaqahabaGaemitaW0aa0baaSqaaiabdohaZjabcYcaSiabigdaXaqaaiabdMgaPbaaaeaacqWGPbqAcqGH9aqpcqaIXaqmaeaacqWGUbGBa0GaeyyeIuoaaOGaayjkaiaawMcaaGGaciab=j7aInaaBaaaleaacqaIXaqmaeqaaOGaey4kaSIaeiOla4IaeiOla4IaeiOla4Iaey4kaSYaaeWaaeaadaaeWbqaaiabdYeamnaaDaaaleaacqWGZbWCcqGGSaalcqWGKbazaeaacqWGPbqAaaaabaGaemyAaKMaeyypa0JaeGymaedabaGaemOBa4ganiabggHiLdaakiaawIcacaGLPaaacqWFYoGydaWgaaWcbaGaemizaqgabeaakiabg2da9maaqahabaGaemOuai1aa0baaSqaaiabdohaZbqaaiabdMgaPbaaaeaacqWGPbqAcqGH9aqpcqaIXaqmaeaacqWGUbGBa0GaeyyeIuoakiabcYcaSaaa@613F@

for *s *= 1, ..., *d*.

We note that the validity of the estimating equation (4) does not depend on any assumption of *d *and *n*. The similarity between the estimating equations (5) and the normal equations for simple linear models motivates variable selection for the additive risk model with right censored data using the following Lasso type estimator:

β^=argminβ{M(β)=∑s=1d{(∑i=1nLs,1i)β1+...+(∑i=1nLs,di)βd−∑i=1nRsi}2},
 MathType@MTEF@5@5@+=feaafiart1ev1aaatCvAUfKttLearuWrP9MDH5MBPbIqV92AaeXatLxBI9gBaebbnrfifHhDYfgasaacH8akY=wiFfYdH8Gipec8Eeeu0xXdbba9frFj0=OqFfea0dXdd9vqai=hGuQ8kuc9pgc9s8qqaq=dirpe0xb9q8qiLsFr0=vr0=vr0dc8meaabaqaciaacaGaaeqabaqabeGadaaakeaaiiGacuWFYoGygaqcaiabg2da9Gqaciab+fgaHjab+jhaYjab+DgaNjab+1gaTjab+LgaPjab+5gaUnaaBaaaleaacqWFYoGyaeqaaOWaaiWabeaacqWGnbqtcqGGOaakcqWFYoGycqGGPaqkcqGH9aqpdaaeWbqaamaacmqabaWaaeWaaeaadaaeWbqaaiabdYeamnaaDaaaleaacqWGZbWCcqGGSaalcqaIXaqmaeaacqWGPbqAaaaabaGaemyAaKMaeyypa0JaeGymaedabaGaemOBa4ganiabggHiLdaakiaawIcacaGLPaaacqWFYoGydaWgaaWcbaGaeGymaedabeaakiabgUcaRiabc6caUiabc6caUiabc6caUiabgUcaRmaabmaabaWaaabCaeaacqWGmbatdaqhaaWcbaGaem4CamNaeiilaWIaemizaqgabaGaemyAaKgaaaqaaiabdMgaPjabg2da9iabigdaXaqaaiabd6gaUbqdcqGHris5aaGccaGLOaGaayzkaaGae8NSdi2aaSbaaSqaaiabdsgaKbqabaGccqGHsisldaaeWbqaaiabdkfasnaaDaaaleaacqWGZbWCaeaacqWGPbqAaaaabaGaemyAaKMaeyypa0JaeGymaedabaGaemOBa4ganiabggHiLdaakiaawUhacaGL9baadaahaaWcbeqaaiabikdaYaaaaeaacqWGZbWCcqGH9aqpcqaIXaqmaeaacqWGKbaza0GaeyyeIuoaaOGaay5Eaiaaw2haaiabcYcaSaaa@7FBA@

subject to the *L*_1 _constraint that

|*β*|_1 _= |*β*_1_| + ... |*β*_*d*_| ≤ *u*,

for a data-dependent tuning parameter *u*, which indirectly determines how many components of β^
 MathType@MTEF@5@5@+=feaafiart1ev1aaatCvAUfKttLearuWrP9MDH5MBPbIqV92AaeXatLxBI9gBaebbnrfifHhDYfgasaacH8akY=wiFfYdH8Gipec8Eeeu0xXdbba9frFj0=OqFfea0dXdd9vqai=hGuQ8kuc9pgc9s8qqaq=dirpe0xb9q8qiLsFr0=vr0=vr0dc8meaabaqaciaacaGaaeqabaqabeGadaaakeaaiiGacuWFYoGygaqcaaaa@2E64@ are zero. With *u *→ ∞, the Lasso estimate will be the same as standard M-estimate, which is not unique in our microarray study. When *u *→ 0, certain components of β^
 MathType@MTEF@5@5@+=feaafiart1ev1aaatCvAUfKttLearuWrP9MDH5MBPbIqV92AaeXatLxBI9gBaebbnrfifHhDYfgasaacH8akY=wiFfYdH8Gipec8Eeeu0xXdbba9frFj0=OqFfea0dXdd9vqai=hGuQ8kuc9pgc9s8qqaq=dirpe0xb9q8qiLsFr0=vr0=vr0dc8meaabaqaciaacaGaaeqabaqabeGadaaakeaaiiGacuWFYoGygaqcaaaa@2E64@ will be exactly zero. Variable selection is achieved since only genes with nonzero estimated coefficients are included in the predictive model. Previous theoretical and practical studies, for example [9] show that with small *u*, the Lasso estimate is shrank towards zero and biased. However with a large number of covariates present, the biased Lasso estimate may have better prediction performance due to the bias-variance tradeoff. This property is especially valuable for microarray data, when a large number of genes are present and many of them are purely noises. In addition, for microarray data analysis, prediction of survival risks and selection of genes are much more important than accurately estimating the coefficients in predictive models.

One unique characteristic of the Lasso estimate in the additive risk model is that the summation in (6) is over *d*, the dimension of covariates, not over the sample size *n *as in the linear regression model. However, considering the equivalence of (6) and (4), the Lasso estimate defined in (6) can provide model reduction in the *β *space. The simplicity of the estimating equation in (6) for the additive risk model is not shared by other survival models. For the Cox model in [9], a weighted least squares approximation to the partial likelihood function and an iterative computational algorithm are needed.

Occasionally, there may exist certain covariate effects that are known to be effective *a priori*. For example, some genes may have been confirmed to be associated with disease occurrence in previous independent studies. In this case interest lies in more accurate adjustment for other gene effects and shrinkage of coefficients (of effective covariates) is not preferred. In such an instance, one may simply omit the corresponding *β_s_*' from the *L*_1 _constraint. The *L*_1 _boosting algorithm discussed below can be applied to such situations with minor modifications.

### Tuning parameter selection

We propose choosing the tuning parameter *u *with the following *V *-fold cross validation [21] for a pre-defined integer *V *. Partition the data randomly into *V *non-overlapping subsets of equal sizes. Chose *u *to minimize the cross-validated objective function

CV score(u)=∑v=1V[M(β^(−v))−M(−v)(β^(−v))],
 MathType@MTEF@5@5@+=feaafiart1ev1aaatCvAUfKttLearuWrP9MDH5MBPbIqV92AaeXatLxBI9gBaebbnrfifHhDYfgasaacH8akY=wiFfYdH8Gipec8Eeeu0xXdbba9frFj0=OqFfea0dXdd9vqai=hGuQ8kuc9pgc9s8qqaq=dirpe0xb9q8qiLsFr0=vr0=vr0dc8meaabaqaciaacaGaaeqabaqabeGadaaakeaacqWGdbWqcqWGwbGvcqqGGaaicqWGZbWCcqWGJbWycqWGVbWBcqWGYbGCcqWGLbqzcqGGOaakcqWG1bqDcqGGPaqkcqGH9aqpdaaeWbqaamaadmaabaGaemyta0KaeiikaGccciGaf8NSdiMbaKaadaahaaWcbeqaaiabcIcaOiabgkHiTiabdAha2jabcMcaPaaakiabcMcaPiabgkHiTiabd2eannaaCaaaleqabaGaeiikaGIaeyOeI0IaemODayNaeiykaKcaaOGaeiikaGIaf8NSdiMbaKaadaahaaWcbeqaaiabcIcaOiabgkHiTiabdAha2jabcMcaPaaakiabcMcaPaGaay5waiaaw2faaaWcbaGaemODayNaeyypa0JaeGymaedabaGaemOvayfaniabggHiLdGccqGGSaalaaa@5B61@

where β^
 MathType@MTEF@5@5@+=feaafiart1ev1aaatCvAUfKttLearuWrP9MDH5MBPbIqV92AaeXatLxBI9gBaebbnrfifHhDYfgasaacH8akY=wiFfYdH8Gipec8Eeeu0xXdbba9frFj0=OqFfea0dXdd9vqai=hGuQ8kuc9pgc9s8qqaq=dirpe0xb9q8qiLsFr0=vr0=vr0dc8meaabaqaciaacaGaaeqabaqabeGadaaakeaaiiGacuWFYoGygaqcaaaa@2E64@^(-*v*) ^is the Lasso estimate of *β *based on the data without the *v*^*th *^subset for a fixed *u *and *M*^(-*v*)^is the function *M *defined in (6) evaluated without the *v*^*th *^subset. When *V *= *n*, we have the widely used Leave-One-Out cross validation. Another possible tuning parameter selection technique is the generalized cross validation as used in [9]. Under mild regularity conditions, all the above cross validation methods are valid and have been shown to have satisfactory numerical results. It is not clear which cross validation method is optimal under the current setting. *V *-fold cross validation with a small *V *is used due to its computational simplicity. We postpone the relative efficacy of different validation techniques to a later study.

### Evaluation

In standard survival analysis with *n *> > *d*, common interest lies in assessing the significance of a covariate via the p-value of its z-score. However, when the sample size is smaller than the number of covariates as in microarray studies, this standard approach of assessing significance may not be appropriate, since its validity typically relies on justifications assuming *n *>> *d*. In addition, in microarray studies, one major goal is to predict survival risks based on selected genes. We propose the following Leave-One-Out (LOO) cross validation based evaluation method for assessing the relative stability of individual genes and overall predictive power.

For *i *= 1, ..., *n*:

1. Generate the reduced dataset by removing the *i*^*th *^subject.

2. Compute the proposed Lasso estimate (including cross validation and estimation) with the reduced set. Denote the estimate of *β *using the reduced set as β^
 MathType@MTEF@5@5@+=feaafiart1ev1aaatCvAUfKttLearuWrP9MDH5MBPbIqV92AaeXatLxBI9gBaebbnrfifHhDYfgasaacH8akY=wiFfYdH8Gipec8Eeeu0xXdbba9frFj0=OqFfea0dXdd9vqai=hGuQ8kuc9pgc9s8qqaq=dirpe0xb9q8qiLsFr0=vr0=vr0dc8meaabaqaciaacaGaaeqabaqabeGadaaakeaaiiGacuWFYoGygaqcaaaa@2E64@^(-*i*)^. Compute the predicted risk score for the *i*^*th *^subject as β^(−i)′Zi
 MathType@MTEF@5@5@+=feaafiart1ev1aaatCvAUfKttLearuWrP9MDH5MBPbIqV92AaeXatLxBI9gBaebbnrfifHhDYfgasaacH8akY=wiFfYdH8Gipec8Eeeu0xXdbba9frFj0=OqFfea0dXdd9vqai=hGuQ8kuc9pgc9s8qqaq=dirpe0xb9q8qiLsFr0=vr0=vr0dc8meaabaqaciaacaGaaeqabaqabeGadaaakeaaiiGacuWFYoGygaqcamaaCaaaleqabaGaeiikaGIaeyOeI0IaemyAaKMafiykaKIbauaaaaGccqWGAbGwdaWgaaWcbaGaemyAaKgabeaaaaa@3565@.

A total of *n *predictive models are generated with the above procedure. For the *s*^*th *^component of *β*, compute the number of times *c*_*s *_it is included in the *n *predictive models (i.e, the number of times that estimated coefficient is not zero). Then the proportion *o*_*s *_= *c*_*s*_/*n *gives a measure of the relative importance and stability of the *s*^*th *^gene. We call *o*_*s *_the occurrence index (OI) of the *s*^*th *^gene. It lies between 0 and 1. Loosely speaking, if a gene is strongly associated with the survival outcome, it should be identified in the reduced datasets. So the corresponding OI should be equal or close to 1. Generally speaking, higher OI indicates more stable and relatively more important gene.

For evaluation of overall predictive power, we dichotomize the *n *predictive risk scores β^(−i)′Zi
 MathType@MTEF@5@5@+=feaafiart1ev1aaatCvAUfKttLearuWrP9MDH5MBPbIqV92AaeXatLxBI9gBaebbnrfifHhDYfgasaacH8akY=wiFfYdH8Gipec8Eeeu0xXdbba9frFj0=OqFfea0dXdd9vqai=hGuQ8kuc9pgc9s8qqaq=dirpe0xb9q8qiLsFr0=vr0=vr0dc8meaabaqaciaacaGaaeqabaqabeGadaaakeaaiiGacuWFYoGygaqcamaaCaaaleqabaGaeiikaGIaeyOeI0IaemyAaKMafiykaKIbauaaaaGccqWGAbGwdaWgaaWcbaGaemyAaKgabeaaaaa@3565@ at their median. Two hypothetical risk groups can then be created. Since different subjects have the same baseline hazard, higher *β*'*Z *leads to higher hazard function and so higher survival risk. So evaluation of prediction (of survival risk) can be constructed based on survival functions of subjects with different predictive risk scores. We compare the difference of survival functions of those two risk groups, which can be measured with a chi-square statistic with degree of freedom one. A significant difference indicates satisfactory prediction performance.

We note that an alternative approach is to create the reduced sets using an approach similar to the V-fold cross validation, i.e, a subset of size *n/V *is removed. The rationale of using the proposed Leave-One-Out approach is that the sample size is usually much smaller than the number of genes for microarray studies. Removing *n*/*V *subjects may leave an even smaller sample size for the reduced data. Estimates from the reduced dataset can be less reliable. The theoretical basis of the proposed evaluation approach is worth further investigation.

### Computational algorithm

The *L*_1 _constraint is equivalent to adding a *L*_1 _penalty to the objective function and ignoring the constraint [9]. Since the *L*_1 _penalty is not differentiable, usual derivative-based minimization techniques (for example the Newton-Raphson) cannot be used to obtain the estimate in (6). In most previous studies, the minimization relies on the quadratic programming (QP) or general non-linear program which are known to be computationally intensive. Moreover, the quadratic programming procedure cannot be applied directly to the settings when the sample size is much smaller than the number of predictors.

Recent study by [22], which relates the minimization step for the Lasso estimate to the *L*_1 _boosting algorithm, a regularized boosting algorithm proposed by [23], provides a computationally more feasible solution. The *L*_1 _boosting algorithm can be applied to general objective functions with *L*_1 _constraints. For the current *L*_1 _constrained estimator defined in (6) with a fixed *u*, this algorithm can be implemented in the following steps:

1. Initialization *β*_*s *_= 0 for *s *= 1 ... *d *and *m *= 0.

2. With the current estimate of *β *= (*β*_1_, ..., *β*_*d*_), compute

φk(β)=∑s=1d{(∑i=1nLs,1i)β1+(∑i=1nLs,di)βd−∑i=1nYsi}×(∑i=1nLs,ki)
 MathType@MTEF@5@5@+=feaafiart1ev1aaatCvAUfKttLearuWrP9MDH5MBPbIqV92AaeXatLxBI9gBaebbnrfifHhDYfgasaacH8akY=wiFfYdH8Gipec8Eeeu0xXdbba9frFj0=OqFfea0dXdd9vqai=hGuQ8kuc9pgc9s8qqaq=dirpe0xb9q8qiLsFr0=vr0=vr0dc8meaabaqaciaacaGaaeqabaqabeGadaaakeaaiiGacqWFgpGzdaWgaaWcbaGaem4AaSgabeaakiabcIcaOiab=j7aIjabcMcaPiabg2da9maaqahabaWaaiWabeaadaqadaqaamaaqahabaGaemitaW0aa0baaSqaaiabdohaZjabcYcaSiabigdaXaqaaiabdMgaPbaaaeaacqWGPbqAcqGH9aqpcqaIXaqmaeaacqWGUbGBa0GaeyyeIuoaaOGaayjkaiaawMcaaiab=j7aInaaBaaaleaacqaIXaqmaeqaaOGaey4kaSYaaeWaaeaadaaeWbqaaiabdYeamnaaDaaaleaacqWGZbWCcqGGSaalcqWGKbazaeaacqWGPbqAaaaabaGaemyAaKMaeyypa0JaeGymaedabaGaemOBa4ganiabggHiLdaakiaawIcacaGLPaaacqWFYoGydaWgaaWcbaGaemizaqgabeaakiabgkHiTmaaqahabaGaemywaK1aa0baaSqaaiabdohaZbqaaiabdMgaPbaaaeaacqWGPbqAcqGH9aqpcqaIXaqmaeaacqWGUbGBa0GaeyyeIuoaaOGaay5Eaiaaw2haaaWcbaGaem4CamNaeyypa0JaeGymaedabaGaemizaqganiabggHiLdGccqGHxdaTdaqadaqaamaaqahabaGaemitaW0aa0baaSqaaiabdohaZjabcYcaSiabdUgaRbqaaiabdMgaPbaaaeaacqWGPbqAcqGH9aqpcqaIXaqmaeaacqWGUbGBa0GaeyyeIuoaaOGaayjkaiaawMcaaaaa@7E85@

for *k *= 1 ... *d*.

3. Find k* that minimizes *min*(*φ*_*k*_(*β*), -*φ*_*k*_(*β*)). If *φ*_k*_(*β*) = 0, then stop the iteration.

4. Otherwise denote *γ *= -*sign*(*φ*_k* _(*β*)). Find α^
 MathType@MTEF@5@5@+=feaafiart1ev1aaatCvAUfKttLearuWrP9MDH5MBPbIqV92AaeXatLxBI9gBaebbnrfifHhDYfgasaacH8akY=wiFfYdH8Gipec8Eeeu0xXdbba9frFj0=OqFfea0dXdd9vqai=hGuQ8kuc9pgc9s8qqaq=dirpe0xb9q8qiLsFr0=vr0=vr0dc8meaabaqaciaacaGaaeqabaqabeGadaaakeaaiiGacuWFXoqygaqcaaaa@2E62@ that

α^
 MathType@MTEF@5@5@+=feaafiart1ev1aaatCvAUfKttLearuWrP9MDH5MBPbIqV92AaeXatLxBI9gBaebbnrfifHhDYfgasaacH8akY=wiFfYdH8Gipec8Eeeu0xXdbba9frFj0=OqFfea0dXdd9vqai=hGuQ8kuc9pgc9s8qqaq=dirpe0xb9q8qiLsFr0=vr0=vr0dc8meaabaqaciaacaGaaeqabaqabeGadaaakeaaiiGacuWFXoqygaqcaaaa@2E62@ = *argmin*_*α*∈[0,1]_*M *[(1 - *α*)(*β*_1_, ..., *β*_*d*_) + *α *× *u *× *γη*_k*_],

where ηk* has the k*^th ^element equals to 1 and the rest equal to 0.

5. Let *β*_*k *_= (1- α^
 MathType@MTEF@5@5@+=feaafiart1ev1aaatCvAUfKttLearuWrP9MDH5MBPbIqV92AaeXatLxBI9gBaebbnrfifHhDYfgasaacH8akY=wiFfYdH8Gipec8Eeeu0xXdbba9frFj0=OqFfea0dXdd9vqai=hGuQ8kuc9pgc9s8qqaq=dirpe0xb9q8qiLsFr0=vr0=vr0dc8meaabaqaciaacaGaaeqabaqabeGadaaakeaaiiGacuWFXoqygaqcaaaa@2E62@)*β*_*k *_for *k *≠ k* and *β*_k* _= (1- α^
 MathType@MTEF@5@5@+=feaafiart1ev1aaatCvAUfKttLearuWrP9MDH5MBPbIqV92AaeXatLxBI9gBaebbnrfifHhDYfgasaacH8akY=wiFfYdH8Gipec8Eeeu0xXdbba9frFj0=OqFfea0dXdd9vqai=hGuQ8kuc9pgc9s8qqaq=dirpe0xb9q8qiLsFr0=vr0=vr0dc8meaabaqaciaacaGaaeqabaqabeGadaaakeaaiiGacuWFXoqygaqcaaaa@2E62@)*β*_k* _+ *γu*α^
 MathType@MTEF@5@5@+=feaafiart1ev1aaatCvAUfKttLearuWrP9MDH5MBPbIqV92AaeXatLxBI9gBaebbnrfifHhDYfgasaacH8akY=wiFfYdH8Gipec8Eeeu0xXdbba9frFj0=OqFfea0dXdd9vqai=hGuQ8kuc9pgc9s8qqaq=dirpe0xb9q8qiLsFr0=vr0=vr0dc8meaabaqaciaacaGaaeqabaqabeGadaaakeaaiiGacuWFXoqygaqcaaaa@2E62@. Let *m *= *m *+ 1.

6. Repeat steps 2–5 until convergence or a fixed number of iterations *N *has been reached.

The *β *at convergence is the Lasso estimate in (6). We conclude convergence if the absolute value of *φ*_k*_(*β*) computed in step 3 is less than a pre-defined criteria, and/or if *M(β) *is less than a pre-defined threshold.

Compared with traditional algorithms, the *L*_1 _boosting only involves evaluations of simple functions. Data analysis experiences show the computational burden for the *L*_1 _boosting is minimal. As pointed out in [22], one attractive feature of the *L*_1 _boosting algorithm is that the convergence rate is independent of the dimension of input. This property of convergence rate is essential to the proposed approach for data like the MCL since the number of genes is very large. On the other hand, it has been known that for general boosting methods, over-fitting usually does not pose a serious problem [24]. So the overall iteration *N *can be taken to be a large number to ensure convergence.

In our numerical study, the above boosting algorithm is realized using R. R code for cross validation, estimation and evaluation is available upon request from the authors. Our empirical study shows that the boosting algorithm is very affordable. For the MCL data, cross validation and estimation combined take less than four minutes.

## Authors' contributions

Both authors are involved in the study design, data analysis and writing. SM wrote the R code for data analysis.
